# A Comparison of Artificial Intelligence and Human Observation in the Assessment of Cattle Handling and Slaughter

**DOI:** 10.3390/ani15091325

**Published:** 2025-05-03

**Authors:** Lily Edwards-Callaway, Huey Yi Loh, Carina Kautsky, Paxton Sullivan

**Affiliations:** Department of Animal Sciences, Colorado State University, Fort Collins, CO 80523, USA; huey.loh@colostate.edu (H.Y.L.); carina.kautsky@colostate.edu (C.K.); paxton.sullivan@colostate.edu (P.S.)

**Keywords:** AI, animal handler, electric prod, fall, stunning efficiency

## Abstract

Slaughter facilities use a variety of tools to evaluate animal handling, including but not limited to live audits, the use of remote video auditing, and some AI technologies. The objective of this study was to determine the similarity between AI and human evaluator assessments of critical cattle handling outcomes in a slaughter plant. One hundred twelve video clips of cattle handling and stunning from a slaughter plant in the United Kingdom were collected. The AI identified the presence or absence of: Stunning, Electric Prod Usage, Falling, Pen Crowding, and Questionable Handling Events. Three human evaluators scored the videos for these outcomes. Four different datasets were generated, and Jaccard similarity indices were generated. There was high similarity (JI > 0.90) for Stunning, Electric Prod Usage, and Falls between the evaluators and the AI. There was high consistency (JI > 0.80) for Pen Crowding. There were differences (JI ≥ 0.50) between the humans and the AI when identifying Questionable Animal Handling Events but the AI was adept at identifying events for further review. The implementation of AI to assist with cattle handling in a slaughter facility environment could be an added tool to enhance animal welfare programs.

## Simple Summary

Animal welfare is a critical component of food animal production. It is important to ensure that animals are provided a good quality of life, up to and including their death. In addition to following governmental regulations for humane handling, companies that slaughter animals also perform internal assessments and often employ the use of technology (e.g., remote video auditing) to enhance their auditing programs. Artificial intelligence (AI) was introduced to enhance the automation of the process and provide more robust oversight (i.e., 24/7 vs. ad hoc). The use of AI to evaluate cattle handling outcomes was compared to the use of human observation to provide insights regarding the accuracy of using AI systems. The results of this project demonstrated that AI could effectively identify cattle handling outcomes such as falling, stunning, and electric prod usage. Humans were also able to accurately identify these outcomes. In some instances, particularly related to questionable animal handling events (i.e., unusual or suspicious activity that could be considered egregious), it would be valuable to use a human-enhanced AI approach, as was conducted in the plant that participated in this study. Artificial intelligence technology could be implemented in slaughter plants to enhance animal welfare evaluation.

## 1. Introduction

Ensuring animal well-being is a core component of production animal management systems, from farm to slaughter. The assessment of an animal’s quality of life must take into account its welfare during all life stages, up to and including the manner of its death [1]. Therefore, animal handling at slaughter plants is a critical factor in maintaining acceptable standards of welfare. Over sixty-nine million tonnes of meat from cattle alone are produced annually across the globe [2], and thus the magnitude of the impact made when improving welfare at slaughter at a global scale is vast. Consumers have demonstrated both an interest in (or concern for) food animal welfare (as reviewed by [3,4]) and a willingness to purchase products that promote animal-friendly practices (meta-analysis by [5]). A large international survey of citizens reported that it mattered to the majority of respondents that animals do not suffer during slaughter [6]. Aside from the ethical responsibility and commitment to animal welfare that stakeholders in the slaughter industry have, there are some economic benefits associated with treating animals humanely (e.g., poor meat quality [7]) that can help support process improvements.

There are different government regulations outlining the requirements for how animals should be treated from their arrival at a slaughter plant through processing (e.g., in the United States—The Humane Methods of Slaughter Act, 1978 [8]; in the United Kingdom—the Welfare of Animals (Slaughter or Killing) Regulations, 1995 [9]) that companies must adhere to and are often verified by in-plant governmental inspection. Although the specific regulations are different across regions, generally, they include similar specifications for humane handling and stunning processes; in the case of the United States and the United Kingdom, regulatory equivalency for humane handling has been granted [10]. Additionally, companies often internally verify humane handling using assessments, third-party audits, and sometimes technology-assisted monitoring (e.g., remote video auditing). In the United States, many companies utilize some type of remote video assessment [11], but this is a voluntary practice. In other countries, for example, in England and Scotland, the use of closed-circuit television (CCTV) has become a mandatory requirement in slaughter facilities in areas where there are live animals [12,13]. The ability to extensively monitor animal handling in slaughter facilities continues to grow as a priority for in-plant welfare programming.

Within recent years, the use of artificial intelligence (AI) as a tool has become increasingly popular [14]. There has been significant growth in the integration of AI into healthcare applications, assisting physicians and personnel with aspects of medical imaging and diagnostics, virtual patient care, patient engagement and compliance, and other administrative applications (as reviewed by [15,16,17,18]). One type of AI, known as human activity recognition (HAR), involves the use of AI technology to analyze raw data in the form of video or images, recognizing and identifying certain actions or behaviors [19]. As HAR technology has the capacity to recognize and differentiate between various human actions, it could be a helpful tool when performing internal plant audits of animal handling by providing real-time monitoring of animal outcomes related to health and welfare [20]. The use of AI to assist with evaluations of animal handling and stunning in slaughter plants could enhance auditing systems. Additionally, the use of AI within the auditing process has the potential to significantly increase both the amount of raw data reviewed and the speed at which it is processed [21]. Proper animal handling and stunning are important parts of the slaughter process, as they have many implications for animal welfare, carcass quality, and consumer perceptions. The objective of this study was to compare the use of AI and human evaluators to accurately evaluate cattle handling outcomes in a slaughter plant environment.

## 2. Materials and Methods

Video clips of animals being handled and stunned at a commercial facility as part of the standard facility operating procedures were evaluated; no animals were experimentally manipulated in this project. A waiver was received from the Colorado State University Institutional Animal Care and Use Committee (#6692). The animals were slaughtered in accordance with the rules set forth in The Welfare of Animals (Slaughter or Killing) Regulations 1995, which are based on EU Council Regulation (EC) No. 1099/2009 (SI 1995/731) [9].

### 2.1. The Animals and the Facility

The video files collected for this project were from a small cattle slaughter plant located in the United Kingdom. The plant operates single 15 h shifts and processes approximately 500 animals per day. The cattle population was a diverse mix of culled cows and bulls and fed steers and heifers of various breeds and ages. The cattle were moved through a single-file chute into a knock box, where they were stunned using a handheld penetrating captive bolt gun, followed by exsanguination. The plant monitored animal handling via the use of 24 h AI monitoring using in-plant cameras positioned at multiple locations, from unloading through stunning. The cameras were commercially available models that were suitable for withstanding the rigors of the in-plant environment. The cameras were positioned so as to capture the maximum view of the focal areas (i.e., holding pens, the knock box, the single-file chute, and the unloading dock). Footage was stored on a server physically located at the establishment, and access was managed on a permissioned basis. The video was only stored for a short period of time to use for further AI training and to meet local regulatory requirements, after which point it was deleted.

### 2.2. AI Training

The AI was trained through a rigorous process involving extensive training. Human reviewers analyzed over 7000 h of video footage from in-plant monitoring, identifying animal handling deficiencies as defined in this project; the deficiencies were then reviewed and validated by the plant’s PAACO-(Professional Animal Auditor Certification Organization)certified expert. These video clips were used to train the AI. Once the AI was operational, the AI flagged potential issues, which were then reviewed to refine its accuracy.

### 2.3. Video Sample Selection

A sample of videos was selected for analysis from clips that the computer-vision-based animal welfare AI system had previously identified as either non-compliant or compliant. The video samples were selected randomly by balancing availability, frequency, and location (with priority on availability). Rare non-compliant cases (e.g., Fall, Pen Density, Questionable Handling Events) were included at their maximum available count. Compliant cases were chosen randomly, ensuring a balanced distribution across locations and dates. All of the clips were 5 min in length, and if a video included an animal handling deficiency, it did not always occur at the same time within the clip (i.e., the deficiency was not always present in the middle of the video clip). A total of one hundred twelve video files were created including all of the handling outcomes listed in Table 1. There were a total of 112 video clips used, but some of the handling outcomes could only occur in certain locations explaining the difference in the number of video clips evaluated per outcome. All video files were given a unique identifier that was AI-generated randomly and saved. The order of the files was randomized evenly to ensure a blind design and prevent bias from expectations. A key representing each video’s identifier and the presence or absence of the specific handling events was created.

### 2.4. Human Evaluators

Two human evaluators (Evaluators 1 and 2; 0 years of experience in auditing but experience working with livestock, familiarity with slaughter systems, and experience collecting data in slaughter plants) were trained by an experienced evaluator (Evaluator 3; more than 10 years of experience in auditing) on the definitions of each handling outcome (Table 1). These definitions were the same definitions used to train the AI to identify the handling outcomes. The definition was shared with the evaluators, and a video example of each outcome was shown. The evaluators were also given a pen map and a pen density chart so they could determine the specific space allowances for each pen. They were also provided with information regarding the length of stay of the animals in the pens, as the pen capacity varied by stay length. The presence (1) and absence (0) of events were recorded by the AI and the 3 evaluators. Evaluators 1 and 2 reviewed all videos independently and recorded which handling events were present in each video file. After evaluators 1 and 2 had reviewed all of the videos, their observations were compared against the key by an additional researcher who was not involved in watching the videos. A list of videos for which there was a discrepancy between the AI’s judgment and that of either of the two human observers was generated by the researcher and submitted to Evaluator 3 for review.

### 2.5. Statistical Analysis

Four different datasets were generated for the similarity tests between the results generated by the AI and those generated by the human evaluators, as some events were mutually exclusive of each other (i.e., videos for pen crowding would not have included an instance of effective stunning, etc.). A total of 27, 31, 50, and 45 videos were evaluated for the similarity between AI and human evaluators for Stunning, Electric Prod Usage, Falling, and Pen Crowding, respectively. All videos (n = 112) were included for the comparison of Questionable Handling Event, and No Deficiency. After the generation of all of the datasets, a Jaccard index (JI; [22]) was generated by R statistical software [23] in RStudio version 2024.04.2+764 comparing the AI’s evaluation (“the key”) against the three human evaluator datasets (Evaluator 1, Evaluator 2, or Evaluator 3). The similarity between the AI (A) and human evaluators (B) was measured by the ratio of their intersection to their union. The Jaccard index can be calculated by dividing the number of shared elements between sets by the total number of elements in both sets combined, which can be represented in this notation form:

$$JI\left(A,B\right)=\frac{\left|A\cap B\right|}{\left|A\cup B\right|}$$

where A represents the observation data generated by AI, and B represents the observation data generated by Evaluators 1, 2, or 3. A JI of 1 indicates that the two datasets are identical, while a JI of 0 means they have zero elements in common. The closer the JI to 1, the higher the similarity between two datasets.

## 3. Results

Table 2 shows the Jaccard similarity indexes for all handling outcomes, comparing the individual human observers’ scores with those of the AI. There was perfect similarity (JI = 1) between Evaluator 3 and the AI for Stunning, Electric Prod Usage, and Falls. There was high similarity (a JI > 0.90) for these three handling outcomes between Evaluators 1 and 2 and the AI. There was high consistency (JI > 0.80) between the AI and all of the human evaluators for Pen Crowding and No Deficiency. These two handling outcomes are related, as the human evaluators identified Pen Density violations in a few videos and the AI did not; thus, discrepancies appear in the analysis for both Pen Density and No Deficiency. There were more differences (JI ≥ 0.50) between the AI and the human evaluators when assessing Questionable Animal Handling Events. 

## 4. Discussion

The incorporation of newer technologies in the food production industry has driven improvements in the food production process [24]. Technologies such as automated carcass-splitting equipment [25], meat imaging systems [26], and warehouse management systems [27] have improved the profitability and efficiency at meat processing facilities. For animal welfare assessments, “smart sensors” and certain measuring devices have been used for observational data collection, health measurements, and behavior detection on farms [28]. Animal-based outcomes (e.g., falling, vocalization, bruising, lesions) are measured frequently by humans in slaughter plants, both in practice and in research, to assess animal welfare, but applications of sensor and AI technologies to measure animal-based outcomes in plants are more limited compared to those on farms [29]. In other production animals, for example, broilers and laying hens, more automated technologies are being used in slaughter plant applications, both commercially and in research, such as the measurement of foot pad lesions [30] and keel bone damage [31]. In a systematic review by Voogt et al. [29] on the use of sensors and AI technology to monitor animal welfare on farms and at slaughter, it was reported that meat color, measured using sensor technology, was the only animal-based measure found in their search; their search did not identify any AI applications for measuring animal-based measures at slaughter. One challenge in using robotics and AI in evaluating pre-slaughter animal welfare is its ability (or difficulty) to flag suspicious events due to the variation in the layout of slaughtering facilities and animal management and handling at plants [24,32]. Although AI technologies have been applied to meat processing [33], to the authors’ knowledge, there have been no assessments of the use of AI to monitor animal handling at slaughter. With that being said, AI technology is a potential tool that slaughter plants could use to assist in pre-slaughter animal welfare evaluations. Hence, the incorporation of AI to enhance the detection, decision-making, and calls for action within monitoring systems is important for pre-slaughter animal welfare assessments.

Depending on the company and region-specific requirements and regulations, plants often take a multi-method approach to in-plant auditing, using in-person audits conducted by an employee in addition to other remote auditing technology, often using video; there is the opportunity to incorporate AI to assist employees in animal welfare monitoring. The Meat Institute’s animal handling and stunning audit guide [34] has been adopted in areas across the world as a foundation for welfare assessments in slaughter plants. The way that animal handlers interact with livestock has a direct impact on animal welfare outcomes [35,36,37], both positively and negatively. At a slaughter facility, cattle are being handled by employees with whom they have never interacted in a new environment that may include additional stressors that could impact their welfare, such as unfamiliar animals, limited space, forced movement, and adverse weather conditions [38]. Ensuring that low-stress handling techniques are used to minimize stress is critical. In addition to impacting welfare, poor handling can also have a direct impact on meat quality (i.e., bruising) [39,40]. The monitoring system incorporating AI used in the current study was trained to be able to identify and mark five different outcomes (Stunning, Electric Prod Usage, Falling, Pen Crowding, and Questionable Handling Event) which can serve as indicators of poor animal welfare. In general, in this study, the assessments by the AI and the human evaluators were highly similar for Stunning, Electric Prod Usage, Falling, and Pen Crowding. Although slightly lower in similarity, the AI and human evaluators demonstrated moderate similarity (50% or more) in assessing Questionable Handling Events. The importance and breakdown of each outcome are discussed below.

Electric prods (or goads) are used when handling animals as a last resort when animals refuse to move forward. In pigs, it has been demonstrated that electric prod usage can both increase physiological indicators of stress (e.g., lactate, cortisol) and decrease meat quality [41,42,43]. Most animal handling audits and guidance documents include an acceptable level of prod usage (e.g., prod usage of 25% or less for cattle [34]). Prod usage is counted when the prod touches the animal whether it is energized or not, as this is difficult to determine when observing. Additionally, in many countries, there are specific regulations about where an animal can [9,44] or cannot [8] be prodded. Prodding an animal in a sensitive area (e.g., on the anus, genitals, or face) is also an audit failure according to the Meat Institute tool [34]. In the current study, the evaluators and the AI were aligned and agreed upon instances of prod usage with high similarity. The discrepancy that did exist was due to human error; one event occurred at the start of a clip almost out of the frame, and it was a challenge for one of the evaluators to identify. Although the other evaluators identified this event and there was only one miss, this may be an example of the usefulness of AI, for capturing events that may be more challenging for humans to identify quickly.

Falling is an outcome that can be impacted by both the animal handlers and the facility conditions. Falling usually occurs at a very low frequency; the Meat Institute audit tool threshold is 1% of animals observed falling [34]. In this study, all instances of falling were identified by the AI and the three evaluators. The instances of falling in this experiment occurred in the drive alley. Animals may fall in the single-file chute or the knock box as well. This is generally rather difficult for a live human observer to observe fully due to restrictions on the visibility into a single-file chute or a knock box. Additionally, when conducting live audits, it is important not to impede animal movement, for example, by standing too close to a single-file chute and causing the cattle to balk due to human presence. If appropriate video angles are used, AI may offer a mechanism for evaluating animal handling in areas that are more challenging for humans to observe.

Effective stunning is a critical criterion used to assess animal welfare during the slaughter process. In conventional slaughter (i.e., non-religious slaughter), the expectation is that cattle should be rendered unconscious immediately with one stun, and this is monitored by evaluating the number of animals for which more than one stun is needed to render it unconscious. It should be noted that stunning an animal more than once does not always mean that the animal was stunned ineffectively the first time; stunning operators may feel it appropriate to deliver a precautionary second shot for a variety of reasons (e.g., equipment failure, imperfect placement, or insufficient air pressure). Behavioral observations of the animal (e.g., eye movements, breathing, vocalization, etc.) should be evaluated post-stunning to assess stunning effectiveness. These behaviors can sometimes be a challenge to identify via video due to differences in the camera angle, the knocker position, animal movement, and the speed of the animals being processed, so often, auditors count the number of times an animal is stunned as a proxy for effectiveness. In the current study, there was high similarity between the evaluators and the AI; two evaluators had perfect similarity with the AI. There was one video that one evaluator determined was not a stunning deficiency. 

The space allowance during holding at a plant (i.e., lairage) is often monitored both internally by the facility and through government oversight. Many slaughter regulations do not prescribe specific space allowances but do require animals to have access to water and space to perform specific behaviors, which impact decisions regarding pen density [8,9]. The Meat Institute [34] provides suggested space allocations for different size animals by weight. In this study, although the similarity between the evaluators and the AI was still high, this outcome was more challenging to assess. This assessment required additional information, not just the viewing of the video, in order to make a determination. The length of stay at the plant prior to slaughter (short vs. long stays) and the pen number were needed to reference a pen density chart that the facility used to determine the maximum capacity. With this many steps in the evaluation process, more errors were made. It is critical that both AI and human evaluators are provided with all of the information required to make these assessments accurately. The differences in the evaluations of Pen Crowding impacted the similarity for the No Deficiency category; outside of these discrepancies, in the current sample of videos, the AI did not miss any of the animal handling deficiencies that the humans thought were present.

The most challenging animal handling events for both the AI and the human evaluators were the Questionable Handling Events. The similarity between the AI and the evaluators in this study was moderate; there were some instances for which full agreement was obtained and others for which it was not. The current process used in the facility that employs this AI to monitor animal handling is the AI identifying a potential occurrence of a deficiency and then a human reviewing the footage to make a final determination as to whether or not the incident requires additional follow-up (i.e., whether it shows an act or condition that results in severe harm, suffering, or distress to animals [45]). It should be noted that there were very few video clips that represented Questionable Handling Events in the study sample; the occurrence of these types of events is very rare, and it was challenging to find clips of this kind to evaluate. Pairing the identification of a potential deficiency via AI with a human evaluation is a robust approach to evaluating animal welfare; this is the process used in the facility that participated in this study. When there is even potential concern about animal welfare, it is better to prioritize risk mitigation by expressing heightened vigilance and training the AI to identify these questionable events. Additionally, if a human evaluator reviews the AI-identified clips, this is an opportunity to further train the AI system to identify animal handling events. It is important to use these types of learning opportunities to further train the AI system as the more examples it is exposed to, the more accurate it becomes.

In-person audits of animal handling only provide information about a snapshot of time, even if audits are conducted multiple times per day. The use of AI for this application could greatly increase the amount of time for which animal handling is evaluated; the AI can provide full coverage of the activities occurring at a facility, including times when the plant has fewer people staffed (i.e., overnight). Additionally, the experience of a human auditor can influence the accuracy of animal handling assessments. In the current study, two of the evaluators had limited in-plant experience, and even though they were trained on the process, they had not received the same training as the AI system had. This emphasizes the importance of auditor training. As humans, it is challenging to sometimes remain unbiased when evaluating animals. For example, a study evaluating the agreement between veterinarians, farmers, and livestock drivers in their evaluation of lameness and fitness for transport reported that the agreement was at best moderate between these groups, calling for more training and calibration [46]. There may be some situations in which human evaluations are valuable because employees are able to provide context that AI will not be able to. As noted for the Questionable Handling Event evaluation, pairing human observations with AI technology may be a way to greatly enhance the robustness of a plant’s animal welfare program.

Although AI has many benefits, the use of AI within the auditing process does come with a unique set of challenges. Data privacy and security can be a concern with HAR technology, as AI becomes intertwined with sensitive information. It is recommended that video footage only be kept for a short period of time if it is needed to train the AI and meet any local regulatory requirements and that access is managed on a permissioned basis. Additionally, with variability in the camera angles, backgrounds, and slaughter plant employees performing the task, HAR technology may face challenges involving biases when identifying certain animal handling behaviors [47], which could be overcome through additional model training. These are issues that should be discussed and considered prior to its implementation. Additionally, it is important to utilize the results of AI monitoring to provide constructive feedback to animal handlers so that they may improve their behavior. As with any auditing system, providing benchmarking information on performance is important, but using the information to enhance and improve programs, morale, and culture is equally as critical.

This study was conducted in one plant in the United Kingdom that slaughtered cattle, and therefore its outcomes are limited in their generalizability. It is necessary to conduct future research in plants that have different facility designs, stunning methods, and types and quantities (i.e., processing speeds) of animals to identify the applicability of AI in other slaughter plant scenarios. Additionally, as with using video monitoring, there is a cost of implementation of using AI for monitoring in plants that should be considered when determining the best option for each facility. As noted, it is suggested that AI in this application is used to support human activities and not necessarily replace them, but employees may have concerns, both perceived and real, about implementing AI in this manner. Effective change management is critical in ensuring staff acceptance and buy-in of this solution.

## 5. Conclusions

Artificial intelligence technology is a tool that could enhance animal welfare programs in slaughter plants. This technology allows for continuous monitoring of animal handling, which is limited when only audits facilitated by employees or third parties are conducted. If properly trained, AI can provide consistent and efficient feedback on animal handling activities that can enhance detection and decision-making. The AI and human evaluators were highly similar in their evaluation of most of the key welfare indicators included in this study. The evaluation of questionable animal events was the biggest challenge, and pairing AI identification of these events with human evaluations could be beneficial. Slaughter plants implement many innovative technologies to enhance their efficiency and performance. AI could serve as another tool to be considered when looking for ways to improve animal welfare programming in slaughter facilities, but practical challenges such as cost and implementation barriers should be considered. Future work should explore the applicability of AI to monitor animal handling in other plant types (i.e., facilities, stunning) in other regions of the world.

## Figures and Tables

**Table 1:** Definitions of outcomes evaluated by both AI and human observers.

Outcome	Definition and Additional Information
Stunning	An effective stun, as defined by the facility, was when only one stun was administered. The video was recorded as rea deficiency when more than one stun was administered to the animal. This was evaluated in the knock box.
Electric Prod Usage	A video was recorded as documenting a deficiency when an animal handler touched an animal with an electric prod. This was evaluated in the single-file chute leading up to the knock box.
Falling	A fall was defined as when an animal loses an upright position suddenly in which a part of the body other than the limbs touches the ground. The video was recorded as a deficiency if this event was present. This was evaluated in the pens, the drive alley, and the unloading area.
Pen Crowding	To make this assessment, both a map of the pen and information regarding the length of stay (short, <3 h, vs. long, >3 h) were needed, as there were different pen capacities depending on these factors. The video was recorded as a deficiency if the animal limit was exceeded for any of the pens in view during the video. This was evaluated in the holding pens.
Questionable Handling Event	The video was recorded as a deficiency if there was an event present that included unusual or suspicious activity that could be considered egregious (e.g., a flagrant or intentional violation of animal welfare), such as hitting an animal with a gate, kicking an animal, or prodding it in a sensitive area. This was evaluated in all of the videos.
No Deficiency	If none of the above-listed outcomes were present in the video, it was considered compliant and recorded as “No Deficiency”. This was evaluated in all of the videos.

**Table 2:** Jaccard similarity indexes of presence (1) and absence (0) of handling outcomes recorded by AI against those of three evaluators. There were a total of 112 video clips used, but some handling outcomes could occur in only certain locations, explaining the difference in the number of video clips evaluated per outcome.

Handling Outcome	# of Videos Included in Analysis	Evaluator 1 ^1^	Evaluator 2 ^1^	Evaluator 3 ^2^
Stunning	27	1.00	0.92	1.00
Electric Prod Usage	31	0.96	1.00	1.00
Falling	50	1.00	1.00	1.00
Pen Crowding	45	0.88	0.83	0.88
Questionable Animal Handling Event	112	0.50	0.50	0.80
No Deficiency	112	0.88	0.88	0.94

^1^ Evaluators 1 and 2 = researchers with 0 years of experience in auditing, trained by an experienced auditor prior to the review of videos. ^2^ Evaluator 3 = a researcher with more than 10 years of experience in auditing.

## Data Availability

The dataset is available on request from the authors.
